# 15-LOX-1 suppression of hypoxia-induced metastatic phenotype and HIF-1*α* expression in human colon cancer cells

**DOI:** 10.1002/cam4.222

**Published:** 2014-03-15

**Authors:** Yuanqing Wu, Fei Mao, Xiangsheng Zuo, Micheline J Moussalli, Elias Elias, Weiguo Xu, Imad Shureiqi

**Affiliations:** 1 Department of Clinical Cancer, The University of Texas MD Anderson Cancer CenterHouston, Texas, 77030; 2 Department of Gastrointestinal Medical Oncology, The University of Texas MD Anderson Cancer CenterHouston, Texas, 77030; 3 Department of Pathology, The University of Texas MD Anderson Cancer CenterHouston, Texas, 77030

**Keywords:** 15-Lipoxygenase-1, angiogenesis, HIF-1*α*, hypoxia

## Abstract

The expression of 15-lipoxygenase-1 (15-LOX-1) is downregulated in colon cancer and other major cancers, and 15-LOX-1 reexpression in cancer cells suppresses colonic tumorigenesis. Various lines of evidence indicate that 15-LOX-1 expression suppresses premetastatic stages of colonic tumorigenesis; nevertheless, the role of 15-LOX-1 loss of expression in cancer epithelial cells in metastases continues to be debated. Hypoxia, a common feature of the cancer microenvironment, promotes prometastatic mechanisms such as the upregulation of hypoxia-inducible factor (HIF)-1*α*, a transcriptional master regulator that enhances cancer cell metastatic potential, angiogenesis, and tumor cell invasion and migration. We have, therefore, tested whether restoring 15-LOX-1 in colon cancer cells affects cancer cells' hypoxia response that promotes metastasis. We found that 15-LOX-1 reexpression in HCT116, HT29LMM, and LoVo colon cancer cells inhibited survival, vascular endothelial growth factor (VEGF) expression, angiogenesis, cancer cell migration and invasion, and HIF-1*α* protein expression and stability under hypoxia. These findings demonstrate that 15-LOX-1 expression loss in cancer cells promotes metastasis and that therapeutically targeting ubiquitous 15-LOX-1 loss in cancer cells has the potential to suppress metastasis.

## Introduction

Metastases are the major cause of death from colon cancer 1. New treatments for colon cancer metastasis are urgently needed; their development will require determination of the critical factors in colon cancer metastasis.

15-Lipoxygenase-1 (15-LOX-1) is an inducible and highly regulated enzyme in normal human cells 2 that plays a key role in the production of important lipid signaling mediators (e.g., 13-S-hydroxyoctadecadienoic acid [13-S-HODE] from linoleic acid 3 and resolvins from eicosapentaenoic acid and docosahexaenoic acid). 15-LOX-1 is important to inflammation resolution 4 and to terminal differentiation of normal cells 2. 15-LOX-1 expression loss is pervasive in cancer cells, as shown in a screen of 128 human cancer cell lines representing more than 20 cancers 5. 15-LOX-1 reexpression in human colon cancer cells by either plasmid or adenoviral vectors induces apoptosis in vitro 6–8 and inhibits xenograft formation in vivo 6,9. Recently, we reported that targeted transgenic 15-LOX-1 expression in the intestine suppresses azoxymethane-induced colonic tumorigenesis 10. 15-LOX-1 is downregulated in various major human cancers (e.g., cancers of the colon 9,11,12, breast 13, lung 5, and pancreas 14). Despite the data supporting the role of 15-LOX-1 as a tumor suppressor gene, current information regarding 15-LOX-1's role in metastasis is limited and conflicting 15. Some data suggest an antimetastatic role for 15-LOX-1. 15-LOX-1 expression is absent in lymph node and liver metastases of pancreatic cancer 14. Levels of 13-S-HODE, the primary product of 15-LOX-1, are inversely associated with cancer cells' ability to attach to endothelial cells and metastasize in mice 16–18. 15-LOX-1 reexpression in colon cancer cells inhibits their invasiveness, motility, and migration in vitro 19,20. Targeted transgenic 15-LOX-1 expression in mouse endothelial cells via the murine preproendothelin-1 promoter markedly inhibits lung metastasis formation by Lewis lung carcinoma cells 21. However, other reports have proposed that 15-LOX-1 promotes metastases. In one report, 15-LOX-1 reexpression in PC-3 prostate cancer cells increased expression of vascular endothelial growth factor (VEGF) in vitro and increased angiogenesis in subcutaneous xenografts 22. In another study, MCF7 breast cancer cells cocultured with immortalized lymphatic endothelial cells to form spheres had higher 15-LOX-1 expression than monolayer cell formations; short hairpin RNA (shRNA) knockdown of 15-LOX-1 reduced MCF7 xenograft formation in mice, and a trend was observed for association between 15-LOX-1 immunohistochemical (IHC) expression in human sentinel lymph node metastases and poor prognosis (*P* = 0.0567) 23. However, findings from the same report called into question the proposed association between 15-LOX-1 and metastases because 15-LOX-1 expression was observed in the weakly invasive MCF7 cells (estrogen receptor positive, epidermal growth factor dependent, luminal epithelial-like) but not in highly invasive fibroblast-like MDA-MB-231 cells (basal-like/triple negative) and because metastasis formation was attributed to 12-hydroxyeicosatetraenoic acid (12-S-HETE), a primary product of 12-S-LOX, but not 13-S-HODE or 15-S-HETE, the primary products of 15-LOX-1 24. Of note, 12-S-HETE and 13-S-HODE have opposing effects on tumorigenesis and metastasis 25. Further studies are, therefore, needed to better define the role of 15-LOX-1 in metastasis.

Hypoxia, a very common feature of the cancer microenvironment, promotes various prometastatic mechanisms (e.g., resistance to cell death, angiogenesis, and tumor cell invasion and migration) 26–28. Hypoxia-inducible factor-1*α* (HIF-1*α*) is a transcriptional master regulator that enhances various metastatic mechanisms (e.g., cell survival, angiogenesis, and invasion) by hypoxia 29 and is upregulated by hypoxia in cancer cells 30,31. HIF-1*α* inhibition or targeted genetic deletion suppresses metastasis in various preclinical models 32,33; therefore, molecular targeting of HIF-1*α* has been pursued 34. Angiogenesis is crucial to the development of metastasis 35,36, and HIF-1*α* promotes several important mechanisms to potentiate tumor angiogenesis via various important proangiogenesis events 37, especially upregulation of VEGF expression 38–40. It is not known whether 15-LOX-1 loss in cancer cells affects cancer cell response to hypoxia, including HIF-1*α* and angiogenesis upregulation and the development of a metastatic phenotype.

We conducted this study to test the hypothesis that restoring 15-LOX-1 in colon cancer cells will inhibit cancer cells' hypoxia response of promoting metastasis and upregulating important events in the pathophysiology of metastasis (e.g., HIF-1*α*, angiogenesis, and tumor cell invasion and migration).

## Material and Methods

### Materials

Monoclonal antibody against HIF-1*α* was obtained from BD Biosciences (San Jose, CA). Methylthiazolyldiphenyl-tetrazolium bromide (MTT) was purchased from Sigma-Aldrich (St. Louis, MO). The human colorectal cancer cell lines HCT116 and LoVo were obtained from American Type Culture Collection (ATCC, Manassas, VA). Human umbilical vein endothelial cell (HUVEC) was purchased from Cambrex (Charles City, IA). HT29LMM cells were kindly provided by Dr. Isaiah J. Fidler (The University of Texas MD Anderson Cancer Center). Cobalt chloride (CoCl_2_) and cycloheximide (CHX) were purchased from Sigma-Aldrich. HIF-1*α* and VEGF real-time PCR probes were purchased from Applied Biosystems (Foster City, CA). Other reagents or chemicals were obtained as specified. Modified Ad-htert-15-LOX-1 (Ad-15-LOX-1) and control-modified Ad-htert-luciferase (Ad-luciferase) adenoviral vectors were developed as described previously 6. The HT29LMM cell line was confirmed by short tandem repeat (STR) through the MD Anderson Cancer Center Characterized Cell Line Core Facility.

### Cell culture conditions

Cells were cultured in McCoy's 5A (HCT116) or RPMI-1640 (LoVo and HT29LMM) supplemented media with 10% fetal bovine serum (FBS) and were maintained in 5% CO_2_ at 37°C. The cells were transfected with phosphate buffered saline (PBS) (mock), Ad-15-LOX-1, or Ad-luciferase at a ratio of 1:200 virus particles (Vp) for LoVo and HCT116 and 1:3200 Vp for HT29LMM in the specified cell culture media supplement with 1% FBS. HUVEC was cultured in HUVEC media containing Endothelial Basal Medium-2 basal medium (CC-3156; Lonza, Walkersville, MD) supplement with Endothelial Growth Media–2 SingleQuots (CC-4176; Lonza) and 1% FBS according to the manufacturer's instructions.

### Hypoxic conditioned medium

HCT116, HT29LMM, and LoVo cells were seeded into 100-mm dishes at a density of 2–3 × 10^6^ cells/dish. The medium was then shifted to 1% FBS on the second day, and the cells were transfected with PBS only (mock), Ad-15-LOX-1, or Ad-luciferase at 1:200 Vp for HCT116 or LoVo or at 1:3200 Vp for HT29LMM under hypoxic conditions in a sealed modular incubator chamber (Billups-Rothenberg, Del Mar, CA) flushed with 1% oxygen (O_2_), 5% carbon dioxide (CO_2_), and 94% nitrogen (N_2_). After 48 h of transfection, the media were harvested, centrifuged at 1250 rpm for 5 min at 4°C, and passed through a 0.22-*μ*m filter. These media served as hypoxic mock-conditioned medium, hypoxic 15-LOX-1-conditioned medium, and hypoxic luciferase-conditioned medium.

### Cell viability/survival assay

The growth rates of the colon cancer cells (HCT116, HT29LMM, and LoVo) and HUVECs were determined by MTT assay. (1) HCT116, HT29LMM, and LoVo cells were seeded in 96-well plates at a density of 5 × 10^3^/well. The cells were transfected with mock, Ad-15-LOX-1, or Ad-Luciferase with 1% FBS media and incubated under hypoxic conditions as previously described for 5 days. (2) HUVECs were seeded in 96-well plates at a density of 3 × 10^3^/well with regular HUVEC medium. After the cells were seeded, the regular medium was replaced with 50% hypoxic conditioned media from HCT116 or HT29LMM plus 50% regular HUVEC medium or with 50% 27.0-*μ*mol/L 13-S-HODE in 1% bovine serum albumin (BSA) of RPMI-1640 plus 50% regular HUVEC media on the next day, and an MTT assay was done after 5 days of incubation under hypoxic conditions. Twenty microliters of 5 mg/mL solution of MTT was added to each well, and the cells were incubated at 37°C for 2 h. MTT was reduced by metabolically active cells to insoluble purple formazan dye crystals. One hundred microliters per well of dimethyl sulfoxide (DMSO) was used for the soluble crystals, and the absorbance was read using a spectrophotometer at an absorbance of 570 nm. All experiments were done in at least triplicate.

Survival was defined as a ratio of MTT measured values for the adenoviral transfected cells to mock transfected cells cultured simultaneously under ideal conditions.

### Migration and invasion assays

Migration and invasion assays of HUVECs and the HCT116 and LoVo colon cancer cells were done using an 8-*μ*m pore size 48-multiwell insert system (Cell Biolabs, Inc., San Diego, CA). Briefly, the methods were as follows: (1) 1.25 × 10^5^ of HUVECs/well were plated on top of inserts in 500 *μ*L of hypoxic conditioned media obtained as previously described or in 500 *μ*L of 13.5-*μ*mol/L 13-S-HODE in 1% BSA of RPMI-1640 media; then 0.75 mL of HUVEC media were added to the bottom of the wells. (2) HCT116 and LoVo cells were seeded into 6-well plates at a density of 8 × 10^5^ cells/well. The medium was then shifted to 1% FBS on the second day, and the cells were transfected with PBS only (mock), modified Ad-15-LOX-1, or Ad-luciferase at 1:200 Vp for HCT116 and LoVo under hypoxic conditions for 48 h. Then, the cells were trypsinized and suspended in 1% FBS McCoy (HCT116) or RPMI-1640 (LoVo), and an equal amount of the cells (1.25 × 10^5^/0.5 mL) were plated on top of the insert; 0.75 mL of 10% FBS of McCoy (HCT116) or RPMI1640 (LoVo) were added to the bottom of the wells. All experimental plates were incubated for 48 h under hypoxic conditions. After incubation, cells that did not migrate were scraped from the top compartment, and the cells that migrated through the membrane were fixed and stained using the protocol of the HEMA 3 stain set (Thermo Fisher Scientific, Pittsburgh, PA). Membranes were excised and mounted on a standard microscope slide (Curtin Matheson Scientific, Houston, TX). The migrated cells were counted with a light microscope at ×100 magnification with at least four random individual fields per insert membrane. Similar methods were used for invasion assays, except the cells were placed in the top insert with the insert membrane coated with 100 *μ*L of growth factor-reduced Matrigel diluted to 300 *μ*g protein/mL (BD Biosciences, Bedford, MA). The numbers of invaded cells were stained and counted from at least four random individual fields visualized at ×100 magnification.

### Tube formation assay

To assess the formation of capillary-like endothelial tubes, 96-well plates were coated with 40 *μ*L of a mixture of growth factor-reduced Matrigel (BD Biosciences) and serum-free RPMI-1640 to a final concentration of 4–5 mg/mL and incubated for 15 min at 37°C. 2 × 10^4^ HUVECs were resuspended in 100 *μ*L of appropriately hypoxic conditioned media from HCT116 cells, dispensed onto growth factor-reduced Matrigel-coated wells, and incubated for 12 h. Tubules were quantified by counting the number of connecting network branches (straight cellular segment) between discrete endothelial cell masses^41^. Images were captured and the average network branch number/low power field (number of branches/LPF) was calculated for all branches counted in three random individual fields per well at ×40 magnification. Network branch number counting was performed by a single investigator who was not the person who captured the images, thus ensuring a double-blind quantification method. Each experiment used medium supplemented with 1% FBS as a negative control and HUVEC culture medium as a positive control, and each experiment was repeated three times.

### Real-time RT-PCR

Twenty-four hours after virus transfection under either hypoxic or normoxic conditions, total RNA samples from HCT116, HT29LMM, and LoVo were isolated using TRIzol reagent (Life Technologies, Grand Island, NY). RNA samples were quantified, and 500 ng of total RNA was reverse transcribed into cDNA using an iScript kit (Bio-Rad Laboratories, Hercules, CA). One microliter of cDNA was used to perform real-time PCR and relative expression study as previously described 8.

### VEGF enzyme-linked immunosorbent assay

Hypoxic conditioned media from HCT116, HT29LMM, or LoVo was separately collected as described before. VEGF protein in hypoxic conditioned media was examined using a human VEGF-specific enzyme-linked immunosorbent assay (ELISA) according to the manufacturer's instructions (Quantikine; R&D Systems, Minneapolis, MN).

### Western blot analysis

Protein samples were extracted from HCT116, HT29LMM, and LoVo 48 h after adenovirus transfections (mock, Ad-15-LOX-1, and Ad-luciferase) under either hypoxic or normoxic conditions. Fifty micrograms of the protein samples were separated onto the 7.5% SDS-polyacrylamide gel, and after electrophoresis, the proteins were transferred to a nitrocellulose membrane. The membranes were blocked with 5% milk for 2 h at room temperature and hybridized with anti-15-LOX-1 at 1:4000 or anti-HIF-1*α* antibody at 1:1000 at 4°C overnight. On the second day, the blots were hybridized with the secondary antibody at 1:10,000 for 1 h at room temperature. The blots were analyzed by using Enhanced Chemiluminescence Plus (ECL plus; GE Healthcare, Piscataway, NJ). ImageJ software (NIH, Bethesda, MD) was used to measure band densities of scanned blot images.

### HIF-1*α* protein stability assay

HIF-1*α* protein stability assay was used to determine whether 15-LOX-1 altered the degradation of HIF-1*α* under hypoxia. HCT116 cells were seeded into 100-mm dishes at a density of 3 × 10^6^/dish. The medium was then shifted to 1% FBS on the second day, and the cells were transfected with PBS only (mock), Ad-15-LOX-1, or Ad-luciferase at 1:200 Vp under hypoxic conditions for 48 h as previously described and then exposed to room air in the presence of 10 *μ*g/mL CHX for the indicated times. Protein samples were harvested and examined for HIF-1*α* expression by Western blot analysis.

### Statistical analysis

Comparisons of single-factor experimental conditions for continuous outcome measures were performed using one-way analyses of variance (ANOVA), and Duncan's adjustments were used for all multiple comparisons. *T*-test was used for two-group comparisons. ANOVA were performed on the log-transformed data to accommodate for the normal-distributional assumptions underlying the methods. All tests were two-sided and conducted at a significance level of *P* < 0.05. Data were analyzed using SAS software (SAS Institute, Cary, NC).

## Results

### 15-LOX-1-inhibited colon cancer cell survival under hypoxic conditions

**Figure 1:**
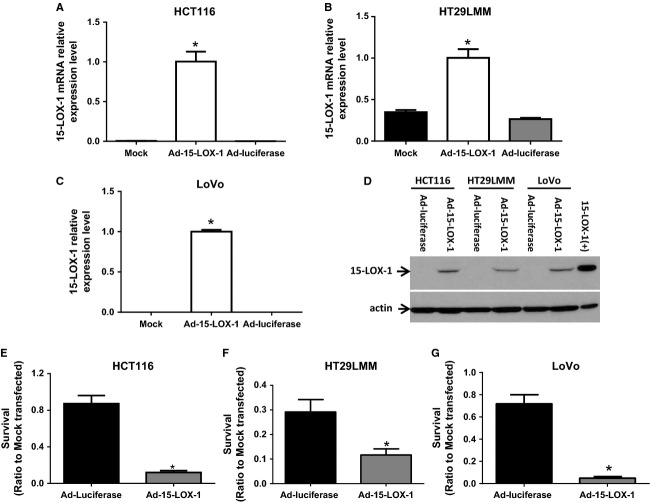
Effect of 15-LOX-1 on colon cancer cell survival under hypoxia. (A–C) The indicated cell lines were transfected with mock, Ad-15-LOX-1, or control Ad-luciferase and cultured under hypoxia for 24 h. Cells were harvested and 15-LOX-1 mRNA levels were measured by real-time RT-PCR for 15-LOX-1. Values are mean ± SD. *P < 0.0001, compared to Mock or Ad-luciferase group, ANOVA. (D) Cells were transfected and cultured as described as panels A–C but for 48 h, and then harvested for 15-LOX-1 protein expression level measurements by Western Blotting. (E-G) The indicated cell lines were transfected with mock, Ad-15-LOX-1, or Ad-luciferase and cultured under hypoxia for 5 days before cell survival (ratio of MTT measured values to mock transfected cells cultured simultaneously under ideal conditions) was measured by MTT assay. Data are presented as ratios to the cells transfected with mock. Values are mean ± SD. *P < 0.0001 compared with the Ad-luciferase group.

Because of hypoxia's important role in activating survival mechanisms in cancer cells that promote metastases 42–45, we examined whether 15-LOX-1 influences colon cancer cell survival under hypoxia. 15-LOX-1 reexpression via Ad-15-LOX-1 in HCT116, HT29LMM, and LoVo colon cancer cells (Fig. 1A–D) markedly suppressed those cells' survival under hypoxia (inhibition ratios of cell survival of Ad-15-LOX-1 to Ad-luciferase: HCT116: 86.34 ± 2.32% (mean ± SD), HT29LMM: 60.08 ± 8.60%, LoVo: 93.18 ± 1.89%) (Fig. 1E–G).

### 15-LOX-1-inhibited angiogenesis and VEGF expression in colon cancer cells

**Figure 2:**
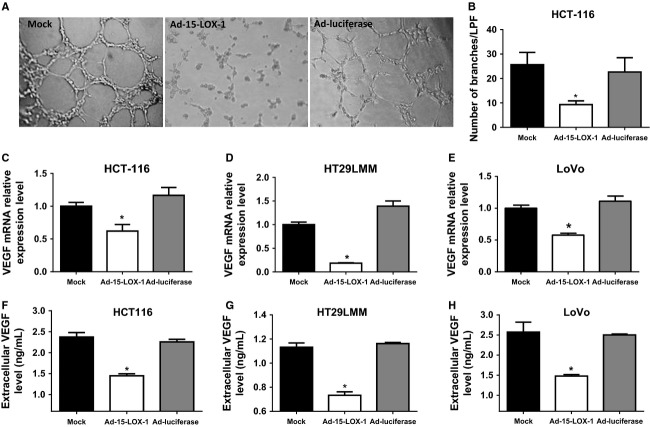
Effect of 15-LOX-1 on angiogenesis and VEGF expression in colon cancer cells. (A) HUVECs were seeded on a Matrigel matrix and incubated with hypoxic conditioned media from colon cancer cells transfected with mock, Ad-15-LOX-1, or Ad-luciferase from HCT116 for 12 h. Capillary tubule formation was examined by inverted light microscopy (magnification ×40). (B) Quantification of effects of 15-LOX-1 on tube formation, as shown in panel A, by averaging the numbers of counted branches in three random fields at ×40 magnification (number of branches/LPF). Results are mean ± SD. *P < 0.02 compared with the Ad-luciferase group, ANOVA. (C–E) Effects of 15-LOX-1 reexpression on VEGF mRNA expressions in colon cancer cells. The indicated cancer cell lines were transfected with Ad-15-LOX-1, Ad-luciferase, or mock, cultured under hypoxia for 24 h, and then harvested. VEGF mRNA expressions were measured by real-time RT-PCR. Values are mean ± SD. *P < 0.001 compared with the Ad-luciferase group, ANOVA. (F–H) Effects of 15-LOX-1 reexpression on VEGF secretion by colon cancer cells. The indicated cancer cell lines were transfected with Ad-15-LOX-1, Ad-luciferase, or mock and cultured under hypoxia for 48 h, and then hypoxic conditioned media were harvested. VEGF protein expressions of hypoxic conditioned media were measured by ELISA method. Values are mean ± SD. *P ≤ 0.0001 compared with the Ad-luciferase group, ANOVA.

Hypoxia causes cancer cells to modify their microenvironment by promoting angiogenesis. We, therefore, examined the effects of 15-LOX-1 reexpression in cancer cells on angiogenesis by comparing tubule formation by HUVECs when incubated for 12 h with that of HCT116 cells transfected with either Ad-15-LOX-1 or Ad-luciferase. The Ad-15-LOX-1-conditioned medium had markedly less HUVEC formation of capillary tubules than the mock or the Ad-luciferase media did (Fig. 2A and B). The number of branches/LPF in the mock (25.67 ± 5.03) was significantly higher than that of Ad-15-LOX-1 (*P* = 0.014, ANOVA) but similar to that of the Ad-luciferase (22.67 ± 5.86).

**Figure 3:**
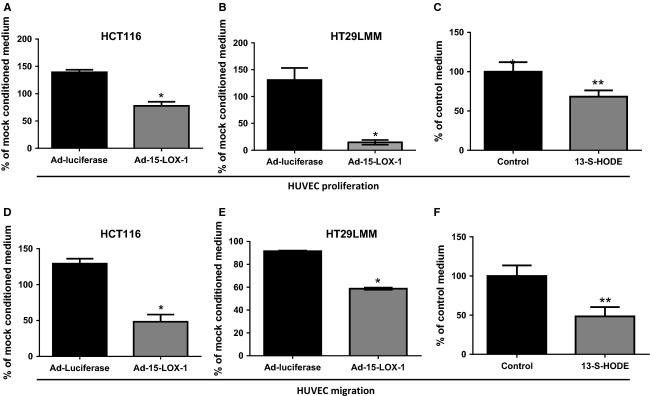
Effects of both 15-LOX-1 and its main product (13-S-HODE) on HUVEC proliferation and migration. (A–C) Effects of 15-LOX-1 reexpression in colon cancer cells and its product 13-S-HODE on endothelial cell proliferation. HUVEC cells were cultured with Ad-Luciferase, Ad-15-LOX-1, or mock-conditioned media from HCT116 or HT29LMM colon cancer cells or cultured with 13.5 *μ*mol/L 13-S-HODE or control media of 13-S-HODE under hypoxia for 5 days. Cell viability was determined by MTT assay. Data are presented as percentage of values of mock-conditioned media for adenoviral transfected cells or control medium for 13-S-HODE treated cells. Values are mean ± SD. *P < 0.0001, **P = 0.005. (D–F) Effect of 15-LOX-1 on HUVEC migration. HUVECs were seeded on top of the insert suspended in Ad-luciferase, Ad-15-LOX-1, or mock hypoxic conditioned media or 13-HODE or control-treated media and cultured under hypoxia. Invaded cells of HUVECs were stained and counted at 48 h after seeding. Data are presented as percentage of values of mock-conditioned media for adenoviral transfected cells or control medium for 13-S-HODE treated cells. Values are means ± SD. *P < 0.0001, **P = 0.001.

We next examined the effects of 15-LOX-1 on VEGF expression in cancer cells given its role as a major proangiogenic factor. 15-LOX-1 reexpression by Ad-15-LOX-1 inhibited VEGF mRNA expression compared to Ad-luciferase control by 47 ± 8% (mean ± SD) in HCT116 cells, 87 ± 0.5% in HT-29LMM cells, and 48 ± 2.5% in LoVo cells (Fig. 2C–E). Ad-15-LOX-1 transfection decreased extracellular VEGF protein levels by 36 ± 2% in the hypoxic culture medium of HCT116 cells, by 37 ± 2.5% in the medium of HT29LMM cells, and by 41 ± 1.5% in the medium of LoVo cells compared to Ad-luciferase transfected cells as measured by ELISA (Fig. 3F–H).

### 15-LOX-1 and its product 13-S-HODE inhibited proliferation and migration of HUVEC cells

We next evaluated the effects of 15-LOX-1 on colon cancer cells altering endothelial cell proliferation and migration, which are enhanced by VEGF expression, to promote angiogenesis. HUVECs incubated with the conditioned medium of HCT116 or HT29LMM cancer cells transfected with Ad-15-LOX-1 had markedly lower proliferation rates than HUVECs incubated with the medium of HCT116 or HT29LMM cancer cells transfected with Ad-luciferase (Fig. 3A and B). We next examined whether 15-LOX-1-modulated endothelial cell migration. HUVECs exposed to the conditioned medium from HCT116 or HT29LMM cells transfected with Ad-15-LXO-1 had reduced migration compared with that of the HUVECs exposed to the conditioned medium of Ad-luciferase transfected HT29LMM (Fig. 3D and E).

Colon cancer cells have reduced levels of 13-S-HODE, the main 15-LOX-1 product, which can repress the survival of these cells when replaced in their culture medium 46. To evaluate whether 13-S-HODE can also influence the tumor microenvironment by modulating endothelial cell proliferation and migration, we added 13-S-HODE to HUVEC culture medium grown under hypoxia. 13-S-HODE reduced HUVEC proliferation by 35 ± 7.5% and HUVEC cell migration by 54 ± 11% compared control medium (Fig. 3C and F).

### 15-LOX-1-inhibited hypoxia promotion of migration and invasion of colon cancer cells

**Figure 4:**
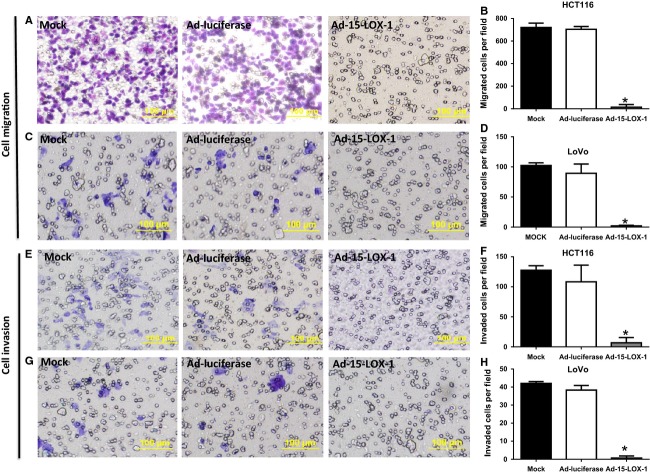
Effect of 15-LOX-1 reexpression on colon cancer cell migration and invasion. (A–H) HCT116 (A–B and E–F) and LoVo (C–D and G–H) cells were seeded and counted for invasion and migration as described in the Methods section. A, C, E, and G are representative photographs of the indicated assays and cell lines. B, D, F, and H are cell counts for the corresponding assays of at least four random individual fields visualized at ×100 magnifications (LPF). Values are mean ± SD. *P < 0.0001.

Hypoxia promotes tumor cell migration and invasion as important mechanisms that confer a metastatic phenotype on cancer cells 26–28. We have, therefore, tested the effects of 15-LOX-1 reexpression in HCT116 and LoVo colon cancer cells on tumor cell migration and invasion under hypoxia. Ad-15-LOX-1 transfection reduced migration of HCT116 by 98 ± 3.4% (mean ± SD) and of LoVo by 98 ± 1.1% compared with Ad-luciferase transfected cells (Fig. 4A–D). Reexpression of 15-LOX-1 by Ad-15-LOX-1 also reduced tumor cell invasion of HCT116 by 94 ± 8.1% and of LoVo by 98 ± 3% compared with the cells transfected with Ad-luciferase (Figs. 4E–H).

### 15-LOX-1 decreased HIF-1*α* expression and increased HIF-1*α* degradation in colon cancer cells

**Figure 5:**
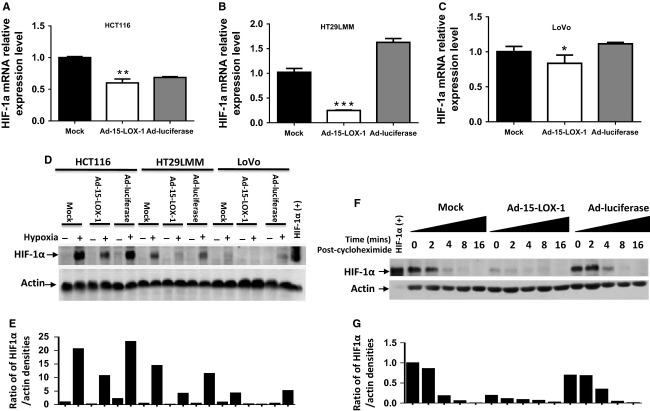
Effect of 15-LOX-1 on HIF-1*α* expression and its stability in colon cancer cells. (A–C) The indicated cell lines were transfected with Ad-luciferase, Ad-15-LOX-1, or mock and cultured under hypoxia for 24 h. Cells were harvested and HIF-1*α* mRNA levels measured by real-time RT-PCR. ***P < 0.0001, **P = 0.0001, *P = 0.02, compared with the Ad-luciferase group. (D–E) Effects of 15-LOX-1 on HIF-1*α* protein expression in colon cancer cells. HCT116, HT29LMM, or LoVo cells were transfected and grown under hypoxia as in panel A–C but for 48 h. Whole cell lysate proteins were analyzed by Western blotting for HIF-1*α*. Positive controls are from HeLa cells treated with 0.4 mmol/L CoCl2 for 4 h under hypoxia. For all three tested cell lines, the Ad-15-LOX-1 group had significantly lower HIF-1*α* protein expression levels than either the mock or the Ad-luciferase group. Repeated experiments showed similar results. (E) Densitometry quantitative analyses of protein bands shown in Figure 5D, values are presented as ratios of HIF-1*α* to actin. (F–G) HCT116 cells were transfected and grown under hypoxia as in panel D and then exposed to room air in the presence of 10 *μ*g/mL cycloheximide for the indicated times. Whole cell lysate proteins were analyzed by Western blotting for HIF-1*α*. For all three tested cell lines, the Ad-15-LOX-1 group had significantly lower HIF-1*α* protein stability than either the mock or the Ad-luciferase group, especially for the first 2 h. Repeated experiments showed similar results. (G) Densitometry quantitative analyses of protein bands shown in Figure 5F, values are presented as ratios of HIF-1*α* to actin.

Because of HIF-1*α*'s established role in promoting various hypoxia-driven prometastatic events (e.g., VEGF expression upregulation, angiogenesis, and tumor cell invasion), we next tested whether 15-LOX-1 modulates HIF-1*α*. 15-LOX-1 reexpression in colon cancer cells reduced HIF-1*α* mRNA to variable degrees. In Ad-15-LOX-1 transfected cells, compared with the corresponding Ad-luciferase transfected cells, HIF-1*α* mRNA expression was suppressed by 12.4 ± 8.87% in HCT116 cells, 84.7 ± 0.5% in HT-29LMM cells, and 25 ± 10.5% in LoVo cells (Fig. 5A–C). Ad-15-LOX-1, however, strongly decreased HIF-1*α* protein expression in all three of those cell lines (Fig. 5D–E). We next tested whether 15-LOX-1 altered the stability of HIF-1*α* under hypoxia. 15-LOX-1 expression strongly increased HIF-1*α* degradation when protein synthesis was inhibited by CHX, especially for the first 2 h (Fig. 5F–G).

## Discussion

We found that 15-LOX-1 reexpression in colon cancer cells suppressed their survival, angiogenesis, cell migration and invasion, and VEGF and HIF-1*α* expression under hypoxia. These data provide needed new insights on the significance of 15-LOX-1 loss in cancer cells with regard to metastasis development.

Our current new findings demonstrate that 15-LOX-1 modulated various important mechanisms for the development of a metastatic phenotype. Hypoxia promotes metastasis through survival mechanisms (e.g., angiogenesis) in response to the harsh microenvironment in rapidly growing tumors 26–28. We have found that 15-LOX-1 markedly inhibited the survival of not only HCT116 cells that were derived from primary colon tumors but also colon cancer cells metastatic origin (LoVo) 47 and those selected in preclinical models for higher metastatic potential (HT29LMM) 48. These findings demonstrate that 15-LOX-1 reexpression in colon cancer cells inhibits their survival not only under normoxic conditions, as we have published previously 6, but also under hypoxic conditions, suggesting that 15-LOX-1 loss in colon cancer cells 15 promotes not only the initial step of tumorigenesis, but also later steps when cells are selected under hypoxia pressure for their metastatic potential.

Our finding that 15-LOX-1 inhibits angiogenesis promotion in colon cancer cell lines further supports the significance of 15-LOX-1 loss for the metastatic phenotype. Angiogenesis is crucial to the progression of tumorigenesis and the development of metastasis 35,36, and one of the best-known tumor proangiogenic factors is VEGF (also referred to as VEGFA) 49. VEGF is upregulated in cancer cells in response to hypoxia, and this upregulation promotes angiogenesis as a mechanism that enhances the metastatic potential of the cells 50. VEGF production by cancer cells, and to a lesser degree by tumor stromal cells, is crucial to tumorigenesis progression 49; anti-VEGF agents such as bevacizumab, a humanized monoclonal antibody directed against VEGF, have been successfully used to treat various human cancers, especially colon cancer 1,51–55. 15-LOX-1's effects on VEGF in cancer cells have been investigated in only one prior study showing that 15-LOX-1 overexpression in the PC-3 prostate cancer cell line increased VEGF expression 22. In contrast, several later studies in various normal cell models showed that 15-LOX-1 inhibited VEGF expression and angiogenesis in noncancerous disease models 56–58. While differences in experimental modeling (e.g., HIF-1*α*, as a VEGF transcriptional driver, is induced by hypoxia in colon cancer cell lines, while PC-3 cells constitutively express HIF-1*α* without hypoxia 59) might have contributed to the contrasting results between our current results in colon cancer cell lines and the prior study of PC-3 prostate cancer cell line, our current results are more consistent with the studies in noncancer models and thus offer the unifying concept that 15-LOX-1 represses VEGF expression and angiogenesis in various disease entities. This concept is further supported by our new findings that 15-LOX-1 expression in colon cancer cells reduced VEGF secretion extracellularly and inhibited proliferation and migration of endothelial cells that were exposed to the media in which the cancer cells were cultured. Supplementing 13-S-HODE to the media of endothelial cells inhibited their proliferation and migration, thus showing the role of 13-S-HODE, a main 15-LOX-1 product, in inhibiting major angiogenesis events. These results demonstrate that 15-LOX-1 reexpression in colon cancer cells modulates cancer cells' ability to modify their microenvironment to promote angiogenesis and subsequently metastasis.

Our novel finding that 15-LOX-1 inhibited HIF-1*α* protein expression in colon cancer cell lines elucidates the mechanisms by which 15-LOX-1 expression in cancer inhibits angiogenesis and metastasis. These findings were confirmed in three colon cancer cell lines and thus are not cell line specific. HIF-1*α* protein expression is upregulated during tumorigenesis via various mechanisms, especially by the reduction in its posttranslational degradation, which increases its stability. For an example, mutational loss of the von Hippel–Lindau protein's ability to bind HIF-1*α* as part of the ubiquitin ligase complex that marks HIF-1*α* for proteasomal degradation increases HIF-1*α* protein levels and promotes renal tumorigenesis 60,61. Another emerging mechanism is posttranslational modification of HIF-1*α* by small ubiquitin-related modifier (SUMO) under hypoxia to initiate ubiquitin-mediated proteasomal degradation of HIF-1*α* 62,63. SUMOylation is regulated by activating enzymes (E1), conjugating enzymes (E2), and ligating enzymes (E3 ligases) and reversed by SUMO-specific isopeptidases (sentrin/SUMO-specific proteases [SENPs]) 64. A key regulator of HIF-1*α* SUMOylation under hypoxia in general, including in cancer cells, is SENP1 62,63. A positive feedback loop exists between SENP1 and HIF-1*α*, as HIF-1*α* directly regulates transcription of the *SENP1* gene 65. Transgenic overexpression of SENP1 in the mouse prostate gland increases HIF-1*α*, VEGF, and angiogenesis 66. Consistent with findings in other cancers 67, SENP1 is overexpressed in human colorectal cancer; targeted reduction in SENP1 inhibits survival of colon cancer cells in vitro and in xenografts 68. In the current study, we showed that while 15-LOX-1 decreased HIF-1*α* mRNA expression to variable degrees and to biologically nonsignificant levels (<30%) in two of three tested cell lines, it consistently decreased protein expression in all tested colon cancer cell lines. Our protein stability analyses further showed that 15-LOX-1 increased HIF-1*α* protein degradation in cancer cells, suggesting that 15-LOX-1 regulates HIF-1*α* at a posttranslational level. The posttranslational mechanisms by which 15-LOX-1 reduced HIF-1*α* protein stability are currently not known and require future studies.

To our knowledge, no prior reports show the effects of 15-LOX-1 on HIF-1*α*. The functional consequences of 15-LOX-1 suppression of hypoxia-induced HIF-1*α* upregulation are demonstrated in findings in this report, showing that 15-LOX-1 also inhibited prometastatic events [expression of VEGF (a HIF-1*α* transcriptional target), angiogenesis, and tumor cell invasion] that are driven by HIF-1*α* secondary to tumor microenvironment hypoxia 26–28; these findings support the role of 15-LOX-1 in suppressing metastases.

In conclusion, our findings support the concept that 15-LOX-1 expression loss in cancer cells promotes not only early stages but also late stages of tumorigenesis, including hypoxia-driven selection of a metastatic phenotype that promotes tumor cell survival, invasion, migration, and ability to modulate the microenvironment via angiogenesis. Our results highlight the significance of 15-LOX-1 repression in later stages of tumorigenesis and the potential development of therapeutic targeting approaches to suppress metastases via reexpression of 15-LOX-1.
